# Cost-Effectiveness of Improving Health Care to People with HIV in Nicaragua

**DOI:** 10.1155/2014/232046

**Published:** 2014-05-25

**Authors:** Edward Broughton, Danilo Nunez, Indira Moreno

**Affiliations:** ^1^USAID Health Care Improvement Project, University Research Co., LLC, Bethesda 20814, USA; ^2^USAID Health Care Improvement Project, University Research Co., Managua, Nicaragua

## Abstract

*Background*. A 2010 evaluation found generally poor outcomes among HIV patients on antiretroviral therapy in Nicaragua. We evaluated an intervention to improve HIV nursing services in hospital outpatient departments to improve patient treatment and retention in care. The intervention included improving patient tracking, extending clinic hours, caring for children of HIV+ mothers, ensuring medication availability, promoting self-help groups and family involvement, and coordinating multidisciplinary care. *Methods*. This pre/postintervention study examined opportunistic infections and clinical status of HIV patients before and after implementation of changes to the system of nursing care. Hospital expenditure data were collected by auditors and hospital teams tracked intervention expenses. Decision tree analysis determined incremental cost-effectiveness from the implementers' perspective. *Results*. Opportunistic infections decreased by 24% (95% CI: 14%–34%) and 11.3% of patients improved in CDC clinical stage. Average per-patient costs decreased by $133/patient/year (95% CI: $29–$249). The intervention, compared to business-as-usual strategy, saved money while improving outcomes. *Conclusions*. Improved efficiency of services can allow more ART-eligible patients to receive therapy. We recommended the intervention be implemented in all HIV service facilities in Nicaragua.

## 1. Introduction

Nicaragua reported 4,742 people living with HIV as of December 2009, 89% of whom were adults over the age of 18 [1]. The epidemic is in an early stage with most cases occurring in high risk populations such as utility workers, commercial sex workers, men who have sex with men, prisoners, street children, housewives, and police and military forces [2]. Antiretroviral therapy (ART) first became available to Nicaraguan patients with HIV early in 2003 and by later that year, there were five health centers in three of the 17 Silais providing such therapy [1]. By 2005, it was reported that ART was available to 35% of those eligible for such treatment [3, 4].

In 2006, the Ministry of Health (MINSA) decided to decentralize provision of HIV services including ART and, in 2009, the USAID Health Care Improvement Project (HCI) was asked to provide technical assistance to attain this goal. HCI worked with MINSA in health centers outside Managua to improve the quality of the services they were providing to HIV patients. As of 2011, 32 out of 34 facilities that dispense ART in all 17 Silais have received technical assistance from HCI. Estimates from epidemiological modeling indicate that about 2,600 patients in Nicaragua are eligible for ART as defined by either clinical stage or CD4+ count [5]. There were just around 1,050 on ART as of June 2009 [6]. In the first half of 2011, MINSA reported 1,442 persons with HIV on ART [7].

A baseline evaluation conducted in January 2009 found that 13% of patients were lost to follow-up and 16.5% were dying while on ART. Only 45% were reported to have good clinical outcomes and 71% of patients were fully compliant with follow-up (unpublished data). To strengthen the system that provides care to HIV patients, HCI with MINSA implemented an intervention to improve the quality of services in seven hospital outpatient departments and two health centers. The goals of the intervention were to improve the quality and continuity of services and thereby improve clinical outcomes among those on ART.

From an analysis of their causes, an intervention was formulated to address problems identified from the baseline assessment with the following components.

### 1.1. Organizational Changes

These include the following:

1. changes to medical records management to ensure availability of important patient data including phone numbers to allow appointment reminders to be sent;
2. reporting of patients who missed appointments to trigger a home visit within 72 hours;
3. extending clinic operating hours to allow access for patients who are unavailable during regular office hours due to their own work;
4. improvements by clinicians in measuring and recording patient data (time on ART, protocol followed, CD4+, viral load, PCR results, drug reactions, opportunistic infections, prophylaxis, and patient's functional status) to allow closer following of patient status and earlier initiation on ART;
5. coordination of all aspects of care in a multidisciplinary team approach;
6. changes to medical care processes to ensure consistent implementation of prevention of mother to child transmission.

### 1.2. Inputs

These include the following:

1. improvement in the supply chain to ensure medication availability;
2. Masaya which was able to provide patients with ambulance services when required.

### 1.3. Psychosocial Support Changes

These include the following:

1. improvements in psychosocial care and support and providing links to self-help groups for patients;
2. counseling for providers to promote more empathic and supportive care;
3. promotion of family support of and involvement in patient care through the use of peer and family counselors.

The objective of this research was to determine how patient status and other indicators of quality of services change using a pre- and postintervention design. The second objective was to determine the cost-effectiveness of the HCI intervention by comparing costs and outcomes between the pre- and postintervention periods.

There have been several evaluations of interventions to improve compliance with ART in terms of their effectiveness [8–10], cost [11], and cost-effectiveness [12] but these are mostly focused on the patient interventions and mostly set in high-income countries. Of those examining provider or facility interventions [13], we found only one that evaluated an adherence improvement strategy in a low-income country setting [14]. In Nicaragua, only one study has examined economic issues of HIV care: a cost analysis of centralized viral load testing [15]. Economic analysis of strategies to improve the quality of care of HIV+ patients on ART is of great importance because health policy officials in Nicaragua and elsewhere are compelled to find the most efficient use of resources for care and treatment, especially given that a high proportion of those needing ART are currently not receiving it.

## 2. Methods

This pre/postintervention study examined the costs and effects of the HCI intervention. There were seven hospitals, all located outside Managua, participating in the improvement intervention from which three facilities were chosen to participate based on the following selection criteria:

1. exposure to at least one year of the improvement intervention;
2. a multidisciplinary team working on the improvement intervention;
3. implementation of all of the changes noted above;
4. no other improvement intervention occurring at the same time or in the last 12 months prior to this intervention.

In these hospitals, patients were sampled based on the following:

1. HIV status confirmed by laboratory status;
2. patient at least aged 18 years;
3. patient on ART at both times of medical record review.

Patients were excluded if they were using different drugs to the national protocol, changed drug regimens during the course of the study, abused drugs or alcohol, had mental disorders, or obtained their drugs through private sources.

Data were collected through three sources: patient medical records for inpatient and outpatient services, hospital expenditure records, and staff costs for following up with patients. A team of three registered nurses and one doctor received training on patient data collection from clinical records. Cost data from the hospital expenditure records were collected by an external data collector and the results were confirmed by hospital administration staff. The four people in each hospital team—the HIV program manager, team coordinator, nurse, and medical assistant in the comprehensive care unit—in all three hospitals involved in following up patients kept a written log of their expenses incurred while following up noncompliant patients in the community.

### 2.1. Analysis

For all changes in patient outcomes between the pre- and postintervention periods, we analyzed the group as a whole and, separately, patients who started ART prior to January 2008. For changes in CDC disease classification [16], we determined the proportion of patients assigned to categories A, A1, A2, B, B1, and B2 (patients without AIDS-indicator infections and with CD4 counts greater than 200 cells/*μ*L) in the two periods and performed Fisher's exact tests for the differences. The same analysis was performed for the proportions of patients with opportunistic infections, at least one hospitalization and CD4 counts below 200/mm^3^. For the total number of opportunistic infections and the total days of hospitalization, Student's* t*-tests were performed.

Decision tree analysis was used to determine the incremental cost-effectiveness from the point of view of the improvement intervention implementers (USAID HCI), MINSA, and health workers using opportunistic infections as the effectiveness outcome. This was because data on the costs of opportunistic infection treatment were available for both in- and outpatient settings and the probability of both was known from patient medical records. The timeframe for the cost-effectiveness analysis was the one-year period of the intervention. Currency was denominated in Nicaraguan Cordobas and converted to 2008 US dollars using the prevailing exchange rate of 20.34 Cordobas to one US dollar [17]. Probabilistic simulations were used to determine a credibility interval around the point estimate for the cost-effectiveness result.

## 3. Results

Most patients were from Chinandega (47%) and Masaya (43%) with Rivas contributing less than 10%. The majority of the patients were male (80%), between ages 25 and 39 (62%), and diagnosed between 2006 and 2007 (62%). More than half started ART before 2008 (Table 1).

For CDC disease classification, we analyzed data in two groups: those who started ART before 2008 and those who started in 2008 or later. Those starting later were more likely to improve due to the effects of being newly prescribed medications to control the HIV disease process. Patients starting earlier were 22% more likely to be in a better clinical stage after the intervention compared to those starting later. Among those starting ART later, their clinical stage was mostly unchanged from the pre- to postintervention periods. Those starting later experienced most improvement in clinical stage before the start of the intervention.

Table 2 shows approximate personal expenses of health staff from the three hospitals spent during follow-up visits and activities for people on ART. The costs paid by nursing staff doing outreach activities as part of the intervention were $15.75 per patient.

Costs to the implementers of the program, HCI, included transportation and other travel expenses, learning sessions, training materials, and technical assistance. The total amount was then divided by the number of patients served by that activity in the hospitals under investigation. Hence the higher* n* of 374 was used for some of the costs. Other expenses were attributable to service delivery in just the three health units included in this study and so were divided by the 133 patient participants (Table 3).

There were statistically significant improvements in the number of patients diagnosed with OIs, the total number of OIs, the number of patients hospitalized, the total number of hospital days, and the proportion of patients with CD4s below 200 from the preintervention to the postintervention period for the group considered together and in the two subsets. Improvements seen in the pre-2008 subset were greater than improvements in the patients starting ART in 2008 or after except for the proportion of patients with CD4 counts below 200 mm^3^.

For the whole group, the risk of opportunistic infections decreased by 24% (95% CI: 14%–34%) from the preintervention period to the end of the intervention. If a patient with HIV did develop an opportunistic infection, the probability of them being treated in hospital rather than in an outpatient setting was about 0.8 in both the preintervention and postintervention periods.

The average per-patient cost of caring for patients decreased by $133/patient/year (95% CI: $29–$249). With this result coupled with the decrease in opportunistic infections, the intervention when compared to the business-as-usual strategy saved money while also improving outcomes.

Probabilistic sensitivity analysis was conducted using the distributions listed in Table 4. For all outcomes, the probability of a cost-saving result with improved outcomes was one.

## 4. Discussion

Results from this study indicate that the intervention to improve care for patients with HIV was associated with improvements in their health as measured by days in hospital, occurrence of opportunistic infections, and CDC clinical stage, while saving money for the health system. This result of the improvement strategy being dominant over the business-as-usual strategy is robust to uncertainties in the input variables. Given that improvements in health are expected in those newly beginning on ART, even in resource-poor settings [18], we separated out those who began ART before 2008 from the whole group. Improvements in the group that started ART earlier were not worse than the group starting later. In fact, those beginning ART earlier had slightly better improvements in hospitalizations and OIs than those starting more recently. Improvements in CD4 counts among those who started ART later were significantly better, a result expected from the effects of beginning ART earlier [18]. The improvements in CD4 counts were not more closely associated with improvements in opportunistic infection, surprising finding given results from other studies comparing CD4 counts with the incidence of such infections [19, 20]. Even allowing those opportunistic infections will still occur in those with higher CD4 counts; those infections should be milder and therefore require shorter average hospitalizations. We did not see that relationship in this study.

The decrease in the period prevalence of opportunistic infections from 38% before the intervention to 14% after the intervention was similar to the decrease in OIs reported from an improvement intervention in San Salvador [21]. These authors found a decrease from 44.9% (95% CI: 34.4 to 55.0%) to 16.9% (95% CI: 9.8% to 26.3%) in the 24 weeks after starting an intervention which included provision of ARTs and prophylaxis, enrollment in self-help groups, education on adherence, and treatment of OIs.

Three studies were found that examined cost-effectiveness of health care improvement interventions for those on ART [14, 22, 23]. They showed that improvement strategies to increase adherence to drug regimens either were cost-saving, as in this study, or had modest costs for substantial benefits.

The decrease in hospitalization due to the lower occurrence of opportunistic infections lowered the overall average cost of caring for patients with HIV by more than one-third. The cost of the intervention associated with this decrease in OIs was $104, of which less than 15% was paid by the unreimbursed activities of the health workers following up on patients missing scheduled visits. For a relatively small initial investment by HCI and MINSA, the health system saved money that could have been used to enroll more HIV positive patients on treatment. Given that other studies from the region have found that starting ART sooner is itself a cost-effective strategy [24], the potential for benefits to MINSA and patients is significant. Therefore, we strongly recommend implementation of this strategy in other parts of the country.

An important component of the costs was the personal expenditures by health workers in the hospital. This contributed about $16 per patient (15%) of the cost of the intervention. Including the expenses due to the intervention borne by providers gave a more complete accounting of the cost of the intervention. However, in an equitable system, these expenses would have been reimbursed by MINSA or HCI. If this intervention is to be considered in the future for other parts of Nicaragua or in other countries, it must be incumbent upon the institution financing the intervention such as the Ministry of Health or outside donor agency, to include these as part of their costs rather than relying on the beneficence of health workers. Securing financing for outreach by health workers is an important factor in securing the sustainability of such interventions [25].

This intervention was conducted in the study sites as sites in the two remote Atlantic Autonomous Regions (RAAN and RAAS after their Spanish acronyms). The considerable costs of participation of health workers from these regions increased the average cost of the intervention for those not in these remote areas. The cost per patient for transportation and other expenses associated with implementation would most likely be considerably lower than the costs in Managua, where distances are much less and the density of patients is much higher. Therefore it is likely that the intervention would be even more attractive from an economic perspective than the result from these remote areas indicates.

### 4.1. Limitations

One significant limitation of the study is the absence of a control group. Had the health changes in patients from participating clinics been compared to those in similar patients from clinics not part of the intervention, the case for attributing the changes seen to the intervention would have been stronger. Limitations on data availability in nonparticipating clinic precluded such a comparison. While no other interventions or major changes aside from those that were part of this program were ongoing during the time of implementation or in the 12 months prior, it cannot be ruled out that other improvements at least a small part in patient status seen could have been due to factors outside the intervention under investigation. For example, if the stigma of being HIV positive decreased universally, this may have increased the proportion of HIV patients remaining on treatment. Any future studies on this topic in this or other settings would be strengthened with use of a control group.

This economic analysis did not take into account the cost consequences to patients of their clinic's participation in the intervention. It is likely that the decrease in opportunistic infections and hospitalization and the improvement in clinical status had a positive economic impact on patients and their families due to their improved overall functioning possibly allowing for more time for employment and other productive economic activities. This has been the case in other studies that have examined cost-effectiveness of treatments to improve the health of patients with HIV [26, 27]. In this study, the factor was not measured because we considered only the perspective of the MINSA, the caregivers, and HCI. Consideration of the societal perspective may have given an even more favorable result.

This analysis was limited to one year of implementation of the intervention. If the improvements in care and the associated improvements in outcomes remained even at a diminished level beyond the period of the intervention, the cost-savings would have been even greater.

## 5. Conclusions

This study demonstrates that an intervention to improve the quality of care for patients on ART in this low-income setting was associated with substantive improvements in health outcomes while decreasing expenditures for that health care. Such improvement in the efficiency of health services can allow more people with HIV in need of ART to receive such therapy. Given this encouraging result, it is recommended that the intervention be implemented in all facilities that deliver HIV services in Nicaragua.

## Figures and Tables

**Table 1:** People with HIV on ART per hospital, gender, and year diagnosed.

	Patients on ART	Percentage
Hospital		
Chinandega	63	47
Masaya	57	43
Rivas	13	10
Sex		
Male	80	60
Female	53	40
Age in years		
19 to 24	15	11
25 to 39	82	62
40 to 59	32	24
60 or more	4	3
Year diagnosed		
Not known	1	1
Before 2003	6	5
2004 to 2007	83	62
2008	42	32
2009	1	1
ART start year		
Before 2008	76	57
2008 or later	57	43

Total	133	

**Table 2:** Personal expenses of health workers in all hospitals.

Cost item	Unit cost US$	Events/month	Months	Total expenses ($US)
Taxi	2.21	10	12	265
Phone	2.95	25	12	885
Food cost	7.87	10	12	944

Total				2094

Annual per patient average cost				**15.75**

Note: exchange rate on January 1, 2009, Cordoba to dollar = C$ 20.34.

**Table 3:** Cost of the improvement collaboration for people with HIV and on ART.

Item	Cost ($US)	Per patient cost
Staff transport for learning sessions (round trip)	798	(*N* = 374)
3 learning sessions with 30 participants, with refreshments	900	
Lodging and dinner for participants from RAAS and RAAN	765	
Air fare for participants from RAAS and RAAN	1998	

Subtotal	**4461**	**11.93**

Training package (standards/indicators manuals and banners)	1026	(*N* = 133)
Technical assistance to health units	9120	

Subtotal	**10146**	**76.29**

Grand total	**14607**	**88.22**

**Table 4:** Cost-effectiveness analysis model inputs.

Opportunistic infection (OI) risk	Estimate	Distribution	95% CI
Preintervention	0.38	Binomial	0.29–0.46
Postintervention	0.14	Binomial	0.08–0.19
Difference	0.24		0.14–0.34
Risk of inpatient hospitalization if diagnosed with an OI			
Preintervention	0.79	Binomial	0.72–0.86
Postintervention	0.83	Binomial	0.77–0.90
Average annual cost of care for person with HIV			
Preintervention	374	Normal	275–485
Postintervention	241	Normal	182–309
Difference	133		29–249
Cost of OI treatment			
Inpatient	1079	Normal	971–1187
Outpatient	687	Normal	618–755

## References

[1] Comision Nicaraguanse del SIDA (2010). *Informe national sobre los progresos realizados en la aplicacion del UNGASS: Nicaragua: Enero 2009-Deciembre 2009*.

[2] Matute AJ, Delgado E, Amador JJ, Hoepelman AIM (2008). The epidemiology of clinically apparent HIV infection in Nicaragua. *European Journal of Clinical Microbiology and Infectious Diseases*.

[3] Comision Nicaraguanse del SIDA (2006). *Informe national sobre los progresos realizados en la aplicacion del UNGASS: Nicaragua: Enero 2003–Deciembre 2005*.

[4] Fajardo R (2009). *Sistematización de impactos y procesos generados en el componente VIH-Sids*.

[5] The Global Fund to Fight AIDS Tuberculosis and Malaria (2011). *Nicaragua Country Statistics*.

[6] The Global Fund to Fight AIDS Tuberculosis and Malaria (2009). *Informe año 5. Proyecto Fondo Mundial: Nicaragua: compromiso y acción ante el SIDA, Tuberculosis y Malaria*.

[7] Ministry of Health (Nicaragua) (2011). *Surveillance Report for HIV/AIDS*.

[8] Andrade ASA, McGruder HF, Wu AW (2005). A programmable prompting device improves adherence to highly active antiretroviral therapy in HIV-infected subjects with memory impairment. *Clinical Infectious Diseases*.

[9] Simoni JM, Pearson CR, Pantalone DW, Marks G, Crepaz N (2006). Efficacy of interventions in improving highly active antiretroviral therapy adherence and HIV-1 RNA viral load: a meta-analytic review of randomized controlled trials. *Journal of Acquired Immune Deficiency Syndromes*.

[10] Tuldrà A, Fumaz CR, Ferrer MJ (2000). Prospective randomized two-arm controlled study to determine the efficacy of a specific intervention to improve long-term adherence to highly active antiretroviral therapy. *Journal of Acquired Immune Deficiency Syndromes*.

[11] Sansom SL, Anthony MN, Garland WH (2008). The costs of HIV antiretroviral therapy adherence programs and impact on health care utilization. *AIDS Patient Care and STDs*.

[12] Zaric GS, Bayoumi AM, Brandeau ML, Owens DK (2008). The cost-effectiveness of counseling strategies to improve adherence to highly active antiretroviral therapy among men who have sex with men. *Medical Decision Making*.

[13] Schneider J, Kaplan SH, Greenfield S, Li W, Wilson IB (2004). Better physician-patient relationships are associated with higher reported adherence to antiretroviral therapy in patients with HIV infection. *Journal of General Internal Medicine*.

[14] Vella V, Govender T, Dlamini SS (2011). Cost-effectiveness of staff and workload profiles in retaining patients on antiretroviral therapy in KwaZulu-Natal, South Africa. *AIDS Care—Psychological and Socio-Medical Aspects of AIDS/HIV*.

[15] Gerlach J, Sequeira M, Alvarado V (2010). Cost analysis of centralized viral load testing for antiretroviral therapy monitoring in Nicaragua, a low-HIV prevalence, low-resource setting. *Journal of the International AIDS Society*.

[16] Centers for Disease Control and Prevention (1992). Extension of public comment period for revision of HIV infection classification system and expansion of AIDS surveillance case definition. *Morbidity and Mortality Weekly Report*.

[17] Oanda Currency converter: historical exchange.

[18] Severe P, Leger P, Charles M (2005). Antiretroviral therapy in a thousand patients with AIDS in Haiti. *The New England Journal of Medicine*.

[19] Deuffic-Burban S, Losina E, Wang B (2007). Estimates of opportunistic infection incidence or death within specific CD4 strata in HIV-infected patients in Abidjan, Côte d’Ivoire: impact of alternative methods of CD4 count modelling. *European Journal of Epidemiology*.

[20] Holmes CB, Wood R, Badri M (2006). CD4 decline and incidence of opportunistic infections in Cape Town, South Africa: implications for prophylaxis and treatment. *Journal of Acquired Immune Deficiency Syndromes*.

[21] Arevalo J, Grande C, Solano Leiva I (2008). Evolución inmunovirológica de adultos infectados por VIH en tratamiento antirretroviral. Experiencia en la unidad médica Atlacatl del ISSS. *Revista Archivos del Colegio Médico*.

[22] Kahn JG, Marseille E, Moore D (2011). CD4 cell count and viral load monitoring in patients undergoing antiretroviral therapy in Uganda: cost effectiveness study. *The British Medical Journal*.

[23] Nachega JB, Mugavero MJ, Zeier M, Vitória M, Gallant JE (2011). Treatment simplification in HIV-infected adults as a strategy to prevent toxicity, improve adherence, quality of life and decrease healthcare costs. *Patient Preference and Adherence*.

[24] Koenig SP, Bang H, Severe P (2011). Cost-effectiveness of early versus standard antiretroviral therapy in HIV-infected adults in Haiti. *PLoS Medicine*.

[25] World Health Organization (2010). *Increasing Access to Health Workers in Remote and Rural Areas Through Improved Retention: Policy Recommendations*.

[26] Bos JM, Postma MJ (2001). The economics of HIV vaccines: projecting the impact of HIV vaccination of infants in Sub-Saharan Africa. *PharmacoEconomics*.

[27] Marseille E, Kahn JG, Pitter C (2009). The cost effectiveness of home-based provision of antiretroviral therapy in rural Uganda. *Applied Health Economics and Health Policy*.

